# Bone-Conducted oVEMP Latency Delays Assist in the Differential Diagnosis of Large Air-Conducted oVEMP Amplitudes

**DOI:** 10.3389/fneur.2020.580184

**Published:** 2020-10-29

**Authors:** Rachael L. Taylor, John S. Magnussen, Belinda Kwok, Allison S. Young, Berina Ihtijarevic, Emma C. Argaet, Nicole Reid, Cheryl Rivas, Jacob M. Pogson, Sally M. Rosengren, G. Michael Halmagyi, Miriam S. Welgampola

**Affiliations:** ^1^Department of Physiology and Center for Brain Research, The University of Auckland, Auckland, New Zealand; ^2^Central Clinical School, Faculty of Medicine and Health, The University of Sydney, Sydney, NSW, Australia; ^3^Macquarie Medical Imaging, Macquarie University Hospital, Sydney, NSW, Australia; ^4^The Balance Clinic and Laboratory, Sydney, NSW, Australia; ^5^Neurology Department and Institute of Clinical Neurosciences, Royal Prince Alfred Hospital, Sydney, NSW, Australia

**Keywords:** vestibular-evoked myogenic potentials, superior semicircular canal dehiscence, tullio phenomenon, vertigo, hyperacusis, bone-conduction

## Abstract

**Background:** A sensitive test for Superior Semicircular Canal Dehiscence (SCD) is the air-conducted, ocular vestibular evoked myogenic potential (AC oVEMP). However, not all patients with large AC oVEMPs have SCD. This retrospective study sought to identify alternate diagnoses also producing enlarged AC oVEMPs and investigated bone-conducted (BC) oVEMP outcome measures that would help differentiate between these, and cases of SCD.

**Methods:** We reviewed the clinical records and BC oVEMP results of 65 patients (86 ears) presenting with dizziness or balance problems who underwent CT imaging to investigate enlarged 105 dB nHL click AC oVEMP amplitudes. All patients were tested with BC oVEMPs using two different stimuli (1 ms square-wave pulse and 8 ms 125 Hz sine wave). Logistic regression and odds ratios were used to determine the efficacy of BC oVEMP amplitudes and latencies in differentiating between enlarged AC oVEMP amplitudes due to dehiscence from those with an alternate diagnosis.

**Results:** Fifty-three ears (61.6%) with enlarged AC oVEMP amplitudes were identified as having frank dehiscence on imaging; 33 (38.4%) had alternate diagnoses that included thinning of the bone covering (near dehiscence, *n* = 13), vestibular migraine (*n* = 12 ears of 10 patients), enlarged vestibular aqueduct syndrome (*n* = 2) and other causes of recurrent episodic vertigo (*n* = 6). BC oVEMP amplitudes of dehiscent and non-dehiscent ears were not significantly different (*p* > 0.05); distributions of both groups overlapped with the range of healthy controls. There were significant differences in BC oVEMP latencies between dehiscent and non-dehiscent ears for both stimuli (*p* < 0.001). A prolonged n1 125 Hz latency (>11.5 ms) was the best predictor of dehiscence (odd ratio = 27.8; 95% CI:7.0-111.4); abnormal n1 latencies were identified in 79.2% of ears with dehiscence compared with 9.1% of ears without dehiscence.

**Conclusions:** A two-step protocol of click AC oVEMP amplitudes and 125 Hz BC oVEMP latency measures optimizes the specificity of VEMP testing in SCD.

## Introduction

Superior canal dehiscence (SCD) is one of several third-mobile window syndromes characterized by an abnormal communication between the inner ear and the intracranial cavity. Diagnosis is best made using a combination of symptoms, CT imaging, and audiovestibular test results, which often includes the recording of vestibular evoked myogenic potentials (VEMPs) (1). VEMP amplitudes are typically enlarged and/or thresholds are reduced as the opening in the superior semicircular canal renders vestibular receptors more susceptible to stimulation by sound and vibration (2–5). Air-conducted (AC) ocular VEMP (oVEMP) amplitudes, cervical VEMP (cVEMP) thresholds, and high frequency (4000 Hz) AC and bone-conducted (BC) oVEMP amplitudes have high sensitivity in discriminating between dehiscent and normal ears (6–9). As AC oVEMP amplitude measurements require fewer trials and minimal departure from standard clinical protocols, they are advocated as the most efficient means of SCD identification (6).

Despite reports of good sensitivity and specificity, most VEMP studies in SCD have been limited to comparisons with healthy controls. However, pathological VEMPs are not specific to SCD. Amplitudes are sometimes enlarged and/or thresholds are reduced in early Meniere's disease (10), enlarged vestibular aqueduct syndrome (11–14) and dehiscence of the posterior canal (15). According to a recent study, false positive (i.e., enlarged) AC oVEMPs may be recorded in around 11% of the non-dehiscent dizzy population (16). Differentiating between SCD and cases of thin bone (near dehiscence) is of particular interest, as there is some suggestion that the latter may be at greater risk of post-operative complications (17). Whether VEMP protocols can be modified to achieve this distinction is currently unclear.

In a previous study, skull-tap oVEMP latencies were identified as an alternative indicator of SCD (18). Tendon hammer taps applied to the upper forehead of patients with SCD produced marked latency delays with sensitivity comparable to AC oVEMP amplitudes. The source of the latency prolongation, which was approximately 4 ms, was hypothesized to be an additional inhibitory inferior oblique muscle response mediated by superior canal afferents. Skull vibration, like changes in intracranial pressure caused by Valsalva (closed glottis) or straining (19), may conduct through the opening in the canal from the soft tissue of the brain and CSF causing additional *ampullopetal* fluid movement. Combinations of ampullopetal and ampullofugal fluid displacement may cause different patterns of otolith and canal receptor activation, producing changes in oVEMP morphology and latency. Intact bone covering should prevent this pressure transference. Thus, we hypothesize the latency effect of bone-conducted (BC) vibration should be specific to SCD.

In this study we investigated whether latency delays produced by a low frequency BC stimulus could assist in differentiating enlarged AC oVEMPs due to dehiscence, from those arising from other pathology. First, we identified cases seen over a five-year period who underwent imaging due to enlarged AC oVEMPs and sought their diagnosis and associated symptoms. BC oVEMP amplitudes and latencies of ears diagnosed with SCD were then compared to those without SCD (i.e., false positive AC oVEMPs) to determine their diagnostic utility.

## Materials and Methods

### Participants

This study was approved by The Royal Prince Alfred Research Ethics and Governance Office. All controls and thirty-two patients provided written consent. Data collected from the remaining patients were used as per existing waiver of consent. All patients with large oVEMPs were studied using this protocol as standard of care.

### Patient Population

Potential participants were identified from the clinical records of neurology clinics at the Royal Prince Alfred Hospital and The Balance Clinic and Laboratory, Sydney Australia. Inclusion criteria required that patients had undergone temporal bone CT imaging at one of two facilities due to enlarged AC oVEMP amplitudes, above the clinical normative range (mean + 2SD of 144 healthy ears; aged 21 to 84 years), *AND* had oVEMP testing with a low-frequency, 125 Hz bone-conducted stimulus. Clinical records of patients meeting these criteria were reviewed for symptom characterization, which was supplemented in most cases by a symptom questionnaire that was administered either face-to-face or over the telephone.

### Healthy Controls

Twenty-one healthy controls (15 female) aged 37.2 ± 9.5 years without vestibular symptoms were recruited for comparison of bone-conduction oVEMP data.

#### Ocular Vestibular Evoked Myogenic Potentials

oVEMPs were recorded using one of two Medelec Synergy evoked-potential systems (software versions 12.2 and 15.0, VIASYS Healthcare UK Ltd). Active (inverting) Ag/AgCl electrodes were placed infra-orbitally beneath the lower lid margin of the contralateral eye, with a reference (non-inverting) electrode placed vertically below it on the cheek. A sternum electrode served as the ground.

**Figure 1:**
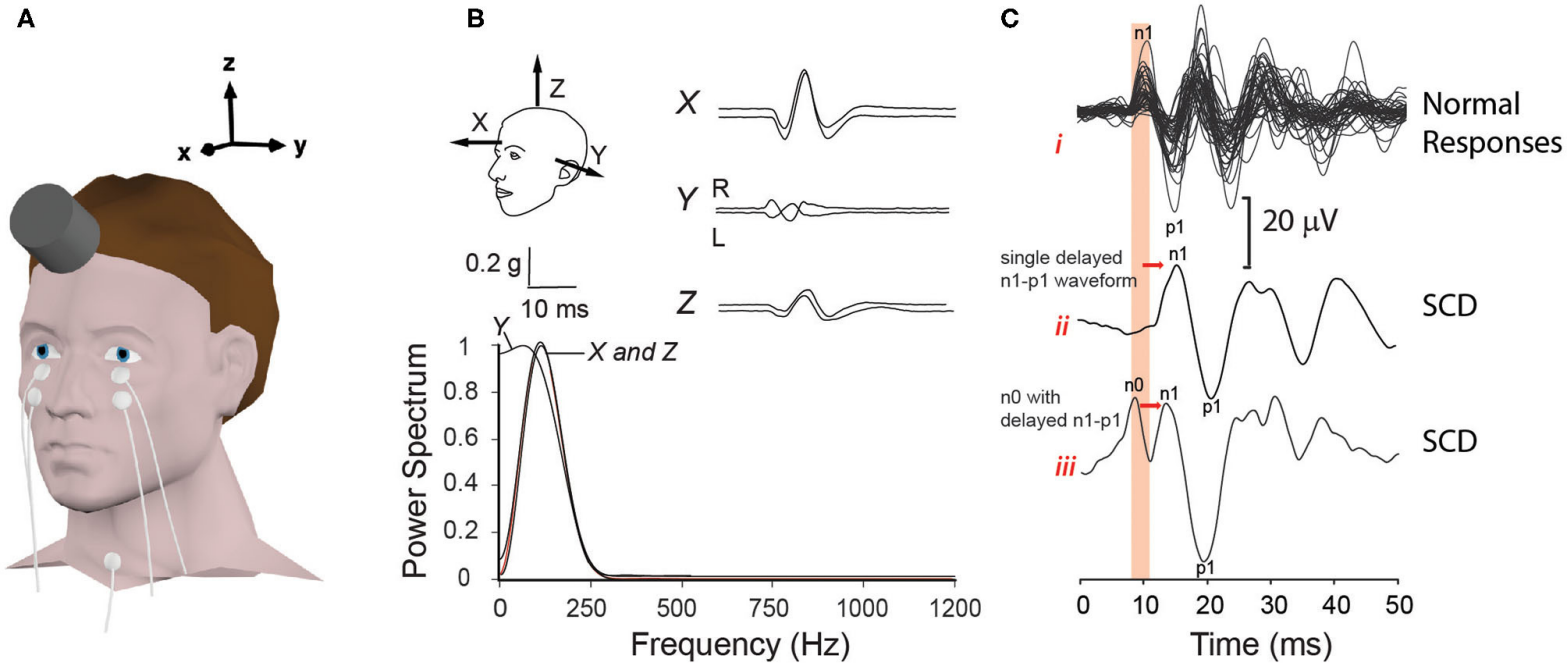
Electrode and transducer details for bone conduction oVEMPs. **(A)** shows the electrode and transducer placement used to record oVEMPs. **(B)** indicates the corresponding three-dimensional (*X, Y* and *Z)* mastoid acceleration (g) response to a 125 Hz sine wave recorded from−10 to +30 ms stimulus onset. Fourier analysis performed over a 10 ms Hanning window, in each axis from stimulus onset, confirms a low frequency power spectrum centered between 125 and 150 Hz. In **(C)**, BC oVEMP waveforms of controls *(i)* are contrasted with two types (*ii* and *iii*) of typical waveforms recorded from SCD patients: *ii* represents a single delayed n1-p1 waveform; in *iii*, the n1-p1 waveform is preceded by an additional up-going n0 potential.

Clinical oVEMP testing was undertaken using three stimuli: 0.1 ms air-conducted clicks (140 dB peak-SPL) delivered with alternating polarity using TDH-49 supra-aural head phones, and bone-conducted vibration consisting of both 1 ms square-wave “minitaps” (MT: 20 V amplitude) and a single cycle 125 Hz sine wave (8 ms duration; 0 ms rise/fall; 20 V peak-peak driving voltage), both with initial condensation polarity. BC stimuli were applied in the midline, close to the hairline (Figure 1A), using a hand-held minishaker (model 4810, Bruel & Kjaer) while the participant lay semi-recumbent. Mastoid accelerometery (Figure 1B) measured for the 125 Hz stimulus from six participants using triaxial accelerometers (TMS international), indicated maximum acceleration in the naso-occipital direction (*X* axis). Fourier analysis of the acceleration response confirmed a low frequency power spectrum, similar to tendon-hammer taps but lower than for minitaps (described previously in 18).

All stimuli were presented at a rate of 5 Hz while the participant gazed upward as high as possible. Responses to 50 (BC) and 100 (AC) stimuli were amplified, band-pass filtered (3–1000 Hz), and averaged. Latencies and peak-to-peak amplitudes for oVEMPs recorded from each ear were determined from markings placed on the first dominant negative-positive (n1-p1) bi-phasic waveform of the contralateral EMG recording (Figure 1C). As in Figure 1C, this was sometimes preceded by an additional smaller negative potential (up-going peak), giving the appearance of a double-peaked negativity. This was coined n0 (18), but as it was not consistently present and the positivity (down-going deflection) between the two n0 and n1 peaks did not cross through zero, it was not analyzed in this study.

#### 3D Temporal Bone Imaging and Patient Classification

Temporal bone imaging was undertaken at two facilities. As a minimum requirement for interpretation, all computerized tomography (CT) imaging included ≤ 0.5 mm cuts reformatted in the plane of all six semicircular canals. CT scans were interpreted by a radiologist with expertise in temporal bone imaging (JM) who was blinded to both the VEMP results and patient symptoms. Scans were classified as N = normal bone covering; 1 = thin bone covering but no dehiscence; 2 = very thin bone covering but no dehiscence; 3 = no visible bone short segment; 4 = no visible bone long segment; 5 = no visible bone; protrudes above tegmen. Imaging results were used to divide patients into two groups. The first group consisted of patients with frank dehiscence (classifications 3–5), representing true positive AC oVEMPs. The second were identified as having intact bone (classifications N, 1 or 2), i.e., false positive AC oVEMPs. Medical records of both groups were reviewed for evidence of alternate or comorbid diagnoses.

### Statistical Analysis

Statistical analysis was performed using SPSS, IBM (version 26) software. Effects of patient group (dehiscent vs. non-dehiscent) and stimulus on oVEMP amplitudes and latencies were compared using a General Linear Mixed Model (GLMM, unstructured covariance), while controlling for age as a covariate. GLMM results are reported in the text as adjusted estimated marginal means and standard errors. Descriptive statistics in the tables represent means (SD). The relationship between individual CT classification scores and oVEMP outcome measures was further explored using Spearman's correlation coefficient. Age adjusted odds ratios (OR) were calculated using binary logistic regression to determine the oVEMP outcome measures and symptoms that were predictive of group membership. Statistical significance was set at *p* < 0.05.

## Results

Sixty-five patients aged 53 ± 13 years (43 female) fulfilled criteria for inclusion in the study. AC oVEMPs were enlarged bilaterally in 21 patients, and unilaterally in 44, comprising a total of 86 ears. None of the cases demonstrated prolonged AC oVEMP n1 latencies. Patient demographics and oVEMP test results are summarized for each CT imaging classification score in Table 1. Fifty-three of 86 ears (61.6%) with enlarged AC oVEMPs (44 patients; 9 bilateral) were diagnosed as dehiscent (classifications 3–5), representing true positive AC oVEMPs. Among the 33 non-dehiscent ears, representing false positive enlarged AC oVEMPs, 13 had near dehiscence (classification 1 or 2). Of the remaining 20 ears (16 patients), 10 patients fulfilled Barany Society criteria (20) for probable or definite vestibular migraine (VM). For four of these patients, VM was the only vestibular diagnosis. Five patients with VM and bilaterally enlarged AC oVEMPs had normal bone-covering on one side and either near or frank dehiscence on the other; another had recovered from a previous episode of vestibular neuritis. A further two patients suffered migraine headaches without fulfilling criteria for VM. Two patients had intractable positional vertigo attributed to BPPV, and another had recurrent spontaneous vertigo of unknown etiology. Enlarged vestibular aqueducts were the probable cause of enlarged AC oVEMP amplitudes in two ears of one patient.

**Table 1:** Patient demographics, VEMP amplitudes and latencies (mean ± SD) summarized for each CT imaging classification score.

**CT Imaging Classification**	**False Positive AC oVEMPs (non-dehiscent ears)**		**True Positive AC oVEMPs (dehiscent ears)**			**Spearman's rho**
	**N**	**1 and 2**	**3**	**4**	**5**	
Number of ears	20 (11F/5M)	13 (11F/2M)	15 (11F/3M)	10 (5F/5M)	28 (16F/8M)	
Age in years	44 ± 13	49 ± 9	59 ± 10	53 ± 10	57 ± 15	
***oVEMP Amplitudes (μV)***						
AC oVEMP	36.3 ± 14.2	44.9 ± 20.4	83.4 ± 30.6	77.1 ± 40.0	58.4 ± 21.7	0.40 (<0.001)
MT oVEMP	47.0 ± 22.6	50.1 ± 21.7	58.2 ± 35.9	65.2 ± 36.5	47.7 ± 18.7	0.01(0.950)
125 oVEMP	41.2 ± 23.5	40.8 ± 22.4	44.7 ± 29.6	41.7 ± 24.3	45.5 ± 22.4	0.07 (0.515)
***oVEMP Latencies (ms)***						
MT oVEMP n1	8.9 ± 0.5	9.5 ± 0.9	10.0 ± 1.1	10.2 ± 0.8	11.1 ± 1.2	0.68 (<0.001)
MT oVEMP p1	13.3 ± 1.1	13.8 ± 1.7	15.3 ± 1.6	15.4 ± 1.2	15.9 ± 1.8	0.59 (<0.001)
125 oVEMP n1	10.0 ± 0.9	10.8 ± 1.4	12.3 ± 1.6	13.1 ± 3.0	13.2 ± 1.9	0.62 (<0.001)
125 oVEMP p1	14.2 ± 0.9	14.9 ± 1.6	16.7 ± 2.0	17.3 ± 3.0	17.6 ± 1.7	0.66 (<0.001)

*The final column represents the strength of the correlation (using Spearman's rho) between each VEMP outcome measure and CT classification score. Some patients with bilaterally enlarged AC oVEMPs are represented in two categories of CT imaging*.

### oVEMP Amplitudes

**Figure 2:**
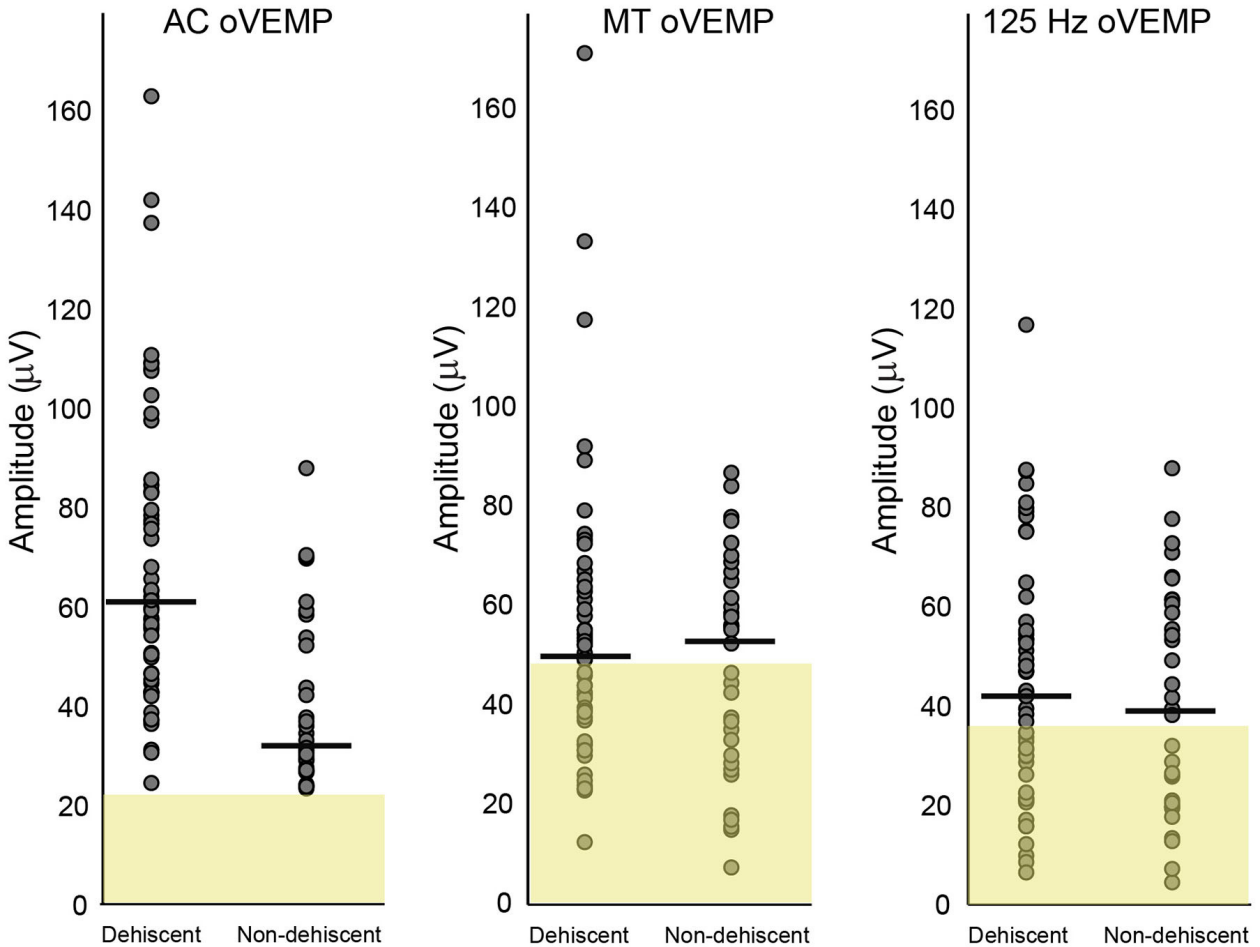
Amplitude comparisons for dehiscent and non-dehiscent ears. Air-conduction oVEMP amplitudes are shown relative to clinical normative data (yellow shaded region) used as recruitment criteria for the study. These data represent the 95% range (mean + 2SD), which defines the upper normal limit as 22.3 microvolts. Normal limits for MT and 125 Hz bone-conduction stimuli represent the mean + 2 SD of the 21 controls recruited in this study, with upper normal limits of 47.6 and 36.2 microvolts, respectively. Horizontal lines indicate group medians.

oVEMP amplitudes for dehiscent and non-dehiscent ears are compared in Figure 2 relative to the clinical normative range (AC stimulus), and results of the 21 control participants (BC stimuli). Analysis of patient results using a GLMM confirmed a significant interaction between stimulus modality and participant group (*F* = 9.438, *p* < 0.001). oVEMP amplitudes in SCD were on average larger in response to AC (70.6 ± 3.5 μV) compared with either BC stimulus (125 Hz = 46.2 ± 3.4 μV; MT = 55.6 ± 3.6 μV), whereas for non-dehiscent patient ears, they were comparable across stimuli. As indicated in Figure 2, amplitude distributions for both BC stimuli overlapped with the range of healthy controls and the odds of either group having an enlarged amplitude above the normal range was not significantly different (MT OR = 1.2, CI: 0.5-3.2, *p* = 0.671; 125 Hz OR = 1.1, 95% CI: 0.4-2.8, *p* = 0.887).

### BC oVEMP Latencies

**Figure 3:**
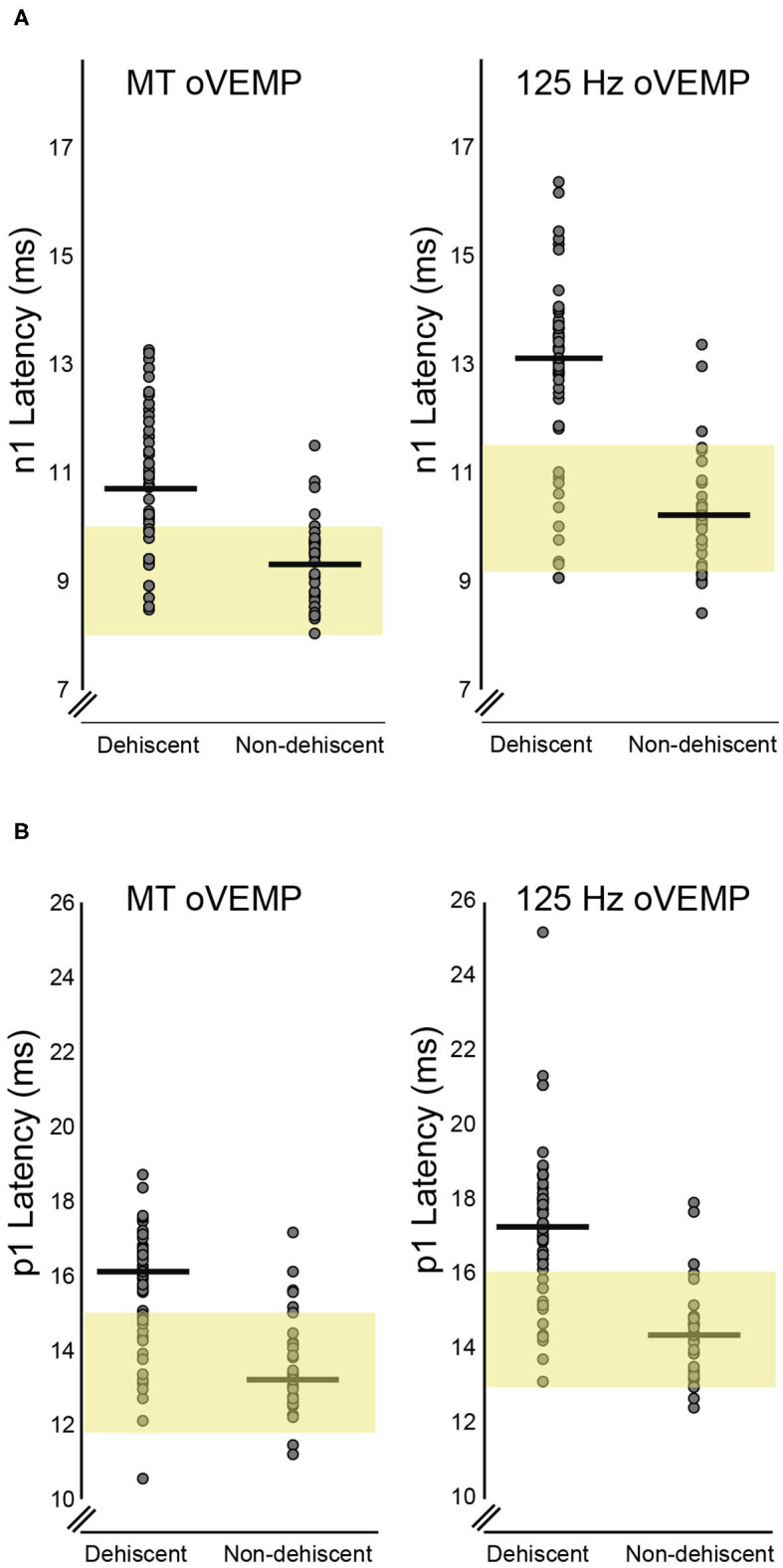
BC Latency comparisons for dehiscent and non-dehiscent ears. **(A)** shows the distribution of n1 latencies for MT and 125 Hz stimuli. Yellow shaded regions correspond to the 95% range (mean +/- 2SD) of values recorded from the 21 controls, which define the upper limit of normal as 10.1 ms for MT and 11.5 ms for 125 Hz stimulation. Median n1 latencies for dehiscent and non-dehiscent ears (horizontal lines) are 10.7 and 9.3 ms for MT and 13.1 and 10.2 ms for 125 Hz. **(B)** shows the latency distributions for p1 potentials relative to the 95% range of control participants. The upper limit of normal for MT and 125 Hz stimulation is defined as 15.0 and 16.1 ms, respectively. Horizontal lines indicate medians of 16.1 and 13.2 ms for dehiscent and non-dehiscent ears for MT stimulation and 17.3 and 14.4 ms for 125 Hz.

Table 2 provides average n1 and p1 latencies for BC stimuli. Compared to the non-dehiscent group, BC oVEMP latencies for n1 and p1 were significantly longer for ears with dehiscence (Figures 3A,B, *p* < 0.001). A significant group by stimulus interaction (*F* = 16.927, *p* < 0.001) further confirmed larger group-mean n1 latency differences for the 125 Hz stimulus (2.3 ± 0.4 ms) than for the MT (1.1 ± 0.2 ms), whereas p1 latency differences for 125 Hz (2.4 ± 0.4 ms) and MT stimuli (1.8 ± 0.4 ms) were more similar (*F* = 3.863, *p* = 0.053). On comparison with the upper normal limits in Figure 3, n1 and p1 abnormality rates for dehiscent ears were 79.2 and 75.5% for 125 Hz and 60.4 and 71.7% for MT. For the non-dehiscent group, n1 and/or p1 latencies were prolonged in 3 of 33 ears (9.1%) for 125 Hz stimulation, all with CT classification scores of 2, and in 5 ears (15.2%) for MT stimulation. Compared with the non-dehiscent group, the odds of a prolonged n1 latency for a patient with dehiscence was 27.8 for 125 Hz (OR 95% CI: 7.0-111.4) and 9.9 for MT stimulation (OR 95% CI: 2.6-38.4). Odds of a prolonged p1 latency was similarly increased by a factor of 21.8 (95%CI: 5.4-87.9) for 125 Hz and 9.4 (95%CI: 3.0-30.0) for MT stimuli.

**Table 2:** Average BC oVEMP amplitudes and latencies for dehiscent, non-dehiscent and control ears.

	**MT**			**125 Hz**		
	**Amplitude**	**n1**	**p1**	**Amplitude**	**n1**	**p1**
Controls	22.6 ± 12.5	9.0 ± 0.6	13.4 ± 0.8	16.1 ± 10.1	10.3 ± 0.6	14.6 ± 0.8
Dehiscence	54.0 ± 28.4	10.6 ± 1.2	15.7 ± 1.6	44.4 ± 24.5	12.9 ± 2.1	17.3 ± 2.1
Non-dehiscence	48.3 ± 22.0	9.1 ± 0.7	13.5 ± 1.4	41.0 ± 22.7	10.3 ± 1.1	14.4 ± 1.3

VEMP results for each CT classification are summarized in Table 1. Moderate positive correlations were evident between all latency measurements and CT scores. There was no relationship between CT scores and either of the BC stimulus amplitudes, but a weak correlation with AC oVEMP amplitudes.

### Contralateral Ears of Patients With Unilaterally Enlarged AC oVEMPs Due to Frank or Near Dehiscence

**Figure 4:**
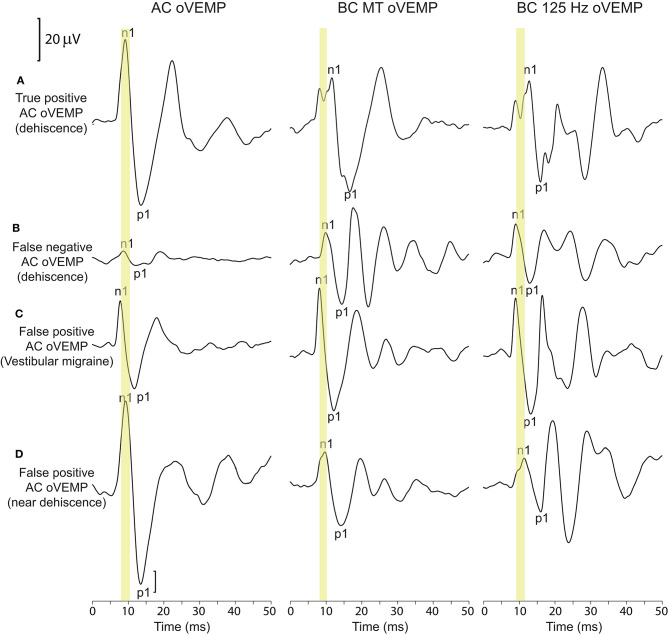
oVEMP waveforms for dehiscent and non-dehiscent ears. **(A, B)** show the corresponding waveforms for the left and right ears of a patient with bilateral dehiscence on CT imaging (classification scores of 4 and 3, respectively). **(A)** represents a true-positive AC oVEMP response. BC oVEMP waveforms show an initial bifid negative potential (described in the methods) with latency delays for both n1 and p1 potentials. The AC oVEMP amplitude in **(B)** falls within normal limits, representing a false negative AC oVEMP; n1 and p1 latencies also fall within the normal range. **(C, D)** show false positive enlarged AC oVEMPs in a patient with vestibular migraine and near dehiscence. BC oVEMP latencies are normal in both cases. The normal n1 latency range is indicated for each stimulus by the yellow shaded region.

CT imaging of the contralateral ears of 35 patients with a positive AC oVEMP and unilateral frank dehiscence revealed 10 cases with normal bone-covering. Sixteen scans revealed thin or very thin bone (classification 1 or 2), three of which were associated with enlarged AC oVEMP amplitudes and were therefore included in the analysis of VEMP results for non-dehiscent ears (Figures 2, 3). A further nine scans were classified as dehiscent despite AC oVEMPs that were either normal or absent (i.e., false *negative* AC oVEMPs), all with a CT classification score of 3 (no visible bone-short segment), normal middle ear function, and normal BC oVEMP latencies. The oVEMP waveforms of a patient with bilateral SCD on CT imaging, showing a false negative AC oVEMP for one ear, are compared in Figure 4 with the waveforms of a patient with VM and near dehiscence.

Near dehiscence *without* enlargement of AC oVEMP amplitudes was also recorded from the contralateral ears of three patients with unilateral enlarged AC oVEMPs due to near dehiscence. Like the nine patients with frank dehiscence and false negative AC oVEMPs, none of these cases had prolonged BC oVEMP latencies.

### Patient Symptoms

Audiovestibular symptoms for dehiscent and non-dehiscent ears are compared in Table 3. For both groups, auditory symptoms were more frequently reported than vestibular symptoms. Conductive hyperacusis, the over-hearing of one's own bodily sounds, was the best auditory discriminator, reported by 74.4% of patients with dehiscence compared with 42.8% without it (OR = 3.9, 95% CI:1.3-11.6, *p* = 0.013). Tullio phenomenon, defined as a positive response to one or more questions relating to sound or pressure induced vertigo/oscillopsia, was experienced by 62.8% of patients with dehiscence compared with 33.3% without dehiscence (OR = 3.4, CI: 1.1-10.1, *p* = 0.027). Based on symptoms and AC oVEMP results, 36 of 43 patients with dehiscence (83.7%)^1^ and 12 of 21 patients without dehiscence (57.1%) fulfilled symptom criteria recommended by the Bárány Society (in press) for a diagnosis of superior canal dehiscence *syndrome* (SCDS). Half the non-dehiscent group fulfilling these criteria had near dehiscence. Spearmen's correlations were performed between the number of SCD-type symptoms (Table 3: questions 1,2,3,4,6,9) and VEMP results for patients with unilaterally enlarged AC oVEMPs. There was no relationship between the number of SCD symptoms and oVEMP amplitudes or n1 latencies in SCD (*p* > 0.3). In contrast, patients without dehiscence who had more symptoms tended to also have larger AC oVEMP amplitudes (rho = 0.583; *p* = 0.047) and longer BC n1 latencies (rho = 0.634, *p* = 0.027; 125 Hz rho = 0.631, *p* = 0.027).

**Table 3:** Prevalence of audio-vestibular symptoms.

	**Dehiscent**		**Non-Dehiscent**	
	**%**	**Sample size (patients/ears)**	**%**	**Sample size (patients/ears)**
**Vestibular symptoms**				
1.*Vertigo-Sound	32.6	43	19	21
2. *Oscillopsia-Sound	25.6	43	19	21
3. *Vertigo-Pressure	48.8	43	28.6	21
4. *Oscillopsia-Pressure	18.6	43	14.3	21
5. Chronic dizziness	45	40	14.3	21
**Auditory symptoms**				
6. Over-hearing of bodily sounds	74.4	43	42.8	21
7. Loudness discomfort	64.4	43	61.9	21
8. Better than normal hearing	25.5	51	40.6	32
9. Autophony	52.0	25	50.0	4
10.Aural fullness	55.7	52	33.3	33
11.Hearing loss	52.0	50	27.3	33

*Percentages indicate the proportion of participants/ears from the total number of available responses (sample size). Percentages for vestibular symptoms, loudness discomfort and hearing of bodily sounds represent the proportion of total patient responses; other auditory symptoms are expressed as a percentage of individual ears. Asterisks indicate symptoms consistent with Tullio phenomenon*.

## Discussion

In this study of 86 ears with enlarged AC oVEMP amplitudes, the most common diagnosis was frank superior canal dehiscence (SCD). All patients with SCD had vestibular and/or auditory symptoms, and 83.7% had symptoms required to fulfill Barany Society criteria for superior canal dehiscence *syndrome* (SCDS). However, enlarged AC oVEMPs were also recorded in association with near dehiscence, vestibular migraine, enlarged vestibular aqueduct, and in a subset of patients without a definitive diagnosis. Half of these cases had symptoms consistent with SCDS. Most ears (79%) with dehiscence demonstrated BC oVEMP latency delays, compared with <16% of ears without dehiscence. These findings support the use of BC oVEMP latency delays in the differential diagnosis of patients with enlarged AC oVEMP amplitudes.

### Delayed BC oVEMPs as a Test of SCD

The findings of this study support the hypothesis that BC oVEMP latency shifts are mediated by a pathological opening in the superior semicircular canal, since they were seen infrequently in other disorders with enlarged AC oVEMP amplitudes. Latency shifts were more pronounced for the 125 Hz stimulus, which is in keeping with previous results using tendon hammer taps which produce a similar low-frequency skull vibration response (18). However, contrasting with results to tendon hammer taps, sensitivity for the 125 Hz stimulus was not 100%. Notably, the sample size in the previous study was smaller and comparisons were made only with healthy controls. Verrecchia et al. (16) similarly found significantly longer Fz 125 Hz latencies for SCD compared with a large group of patients with unselect dizziness, though sensitivity and specificity were lower than for 500 Hz AC oVEMP amplitudes. Whether any of their non-dehiscent patients had both an enlarged AC oVEMP amplitude, and a delayed 125 Hz latency, was not reported. In our study, where an enlarged AC oVEMP was a requirement for inclusion, prolonged 125 Hz n1 latencies >11.5 ms were occasionally recorded in ears with extremely thin bone covering (i.e., near dehiscence). This implies fluid movement through the canal opening is not always necessary. In some cases, flexing of the compliant bone could be sufficient to produce a similar pattern of endolymph displacement and receptor activation, accounting for both the AC oVEMP amplitude enlargement and BC oVEMP latency delay.

### Near Dehiscent Ears

Near dehiscence was the most common alternate cause of enlarged AC oVEMPs in this series. Cadaveric studies indicate a prevalence of ~1.4%, meaning near dehiscence is ~3-fold more common than frank dehiscence (21). As our data suggest, these cases may or may not be associated with enlarged AC oVEMP amplitudes and SCD-type symptoms. Interest in separating near from frank dehiscence arose following the observation of a possible increase in post-operative complications, which included permanent hearing loss, transient facial nerve palsy and recurrence of symptoms (17). In a subsequent case-controlled study there was no difference in the rate of surgical complications. However, enduring post-operative auditory symptoms were documented in 41% of near dehiscence patients as opposed to 18% with frank dehiscence (22). Thus, distinguishing between etiologies could still be helpful in pre-surgical counseling/planning.

Compared with frank dehiscence, oVEMP amplitudes in near dehiscence tend to be lower (22) and cVEMP thresholds higher (22, 23). This was also observed in the present study for comparisons between AC oVEMP amplitudes of dehiscent and non-dehiscent patient ears. However, the overlapping amplitude distributions make it difficult to establish a definitive cut-off without compromising sensitivity and specificity. Even greater overlap was evident between BC oVEMP amplitude distributions, an effect that may be explained by different patterns of endolymph flow, end organ, and receptor activation. Whereas, both stimuli produce combinations of ampullofugal and ampullopetal endolymph pumping and flow (3), eye movement recordings suggest the dominant effect of air-conduction in SCD is otolith and superior canal afferent excitation. The eyes (slow phase) move upward and away from the stimulated ear, reflecting ampullofugal fluid displacement from the oval window of the cochlea toward the dehiscence (24–26).

Low frequency vibration (~100 Hz) is a less specific stimulus for the otolith afferents (27) that can reach the labyrinth through a combination of inertial, compression, and soft-tissue/fluid pathway mechanisms (28). Distributed patterns of fluid displacement and receptor activation across different parts of the labyrinth probably explains the more modest enhancement in BC oVEMPs and BC-evoked eye movements (8, 26), and the more diverse patterns of nystagmus reported in response to low-frequency BC vibration (29–32).

Latency comparisons were significant not only between patient groups, but in comparison with the upper limit of controls, where a prolonged BC oVEMP latency predicted dehiscence. Previous attempts to discriminate between dehiscence and near dehiscence have met with mixed results. Mehta et al. (23) found no significant differences in DHI scores or objective findings of sound or pressure-evoked nystagmus. In contrast, for a group of patients undergoing SCDS surgery, sound or pressure-evoked nystagmus were more common in frank dehiscence (22). cVEMP thresholds and air-bone gaps on audiometry have been advocated as useful discriminators (22, 23), and oVEMP latencies and amplitudes for BC vertex stimulation can help separate SCD from other causes of dizziness (16). However, threshold seeking and vertex oVEMPs require either additional recordings or a shift in BC stimulation site, and not all neurotologists will have ready access to an audiometer.

### Alternate Diagnoses in Patients With Large AC oVEMPs

This study highlights additional diagnoses, other than frank or near dehiscence, that can produce enlarged AC oVEMPs. The finding of two cases with enlarged vestibular aqueducts, another third-window syndrome, is unsurprising and has been described previously (13, 14). Similar to SCD, the enlarged aqueduct creates an additional low impedance pathway, through which sound, vibration and pressure can transmit (33). Absence of a BC oVEMP latency delay in these cases could reflect the different anatomical location of the third window. A low impedance pathway between the aqueduct and cochlear windows (i.e., through the vestibule) could lead to increased otolith hair cell stimulation but without significant fluid displacement and hair cell activation within the superior canal. Further studies involving patients with enlarged vestibular aqueducts are needed to confirm this. No cases of posterior canal dehiscence were identified in this series to determine whether BC oVEMP latency delays occur with increased posterior canal receptor activation.

The finding of enlarged AC oVEMPs in association with VM is more difficult to explain, since there is no third window into the inner ear and VM is a central vestibular disorder (20). VEMP results in VM are variable, ranging from reduced or absent responses (34, 35), to normal responses (36, 37) that sometimes potentiate with repetitive stimulation (38). Potential mechanisms underlying vestibular symptoms and signs are equally diverse and could include any combination of inner ear ischemia due to vasospasm, trigeminal nerve irritation, and central disruptions in sensory processing. In our experience, most VM patients have normal and symmetrical VEMP responses (39). However, just as some VM patients demonstrate hyper-responsivity on caloric testing (40, 41), the enlarged oVEMP responses described herein may represent a subset of patients for whom central mechanisms of vestibular hyperexcitability are dominant. Associated symptoms of aural pressure and hyperacusis (42), further highlight VM as a potential SCD mimic for which adjunct BC oVEMP latency testing could prove useful.

### False Negative oVEMPs

This study revealed nine incidental cases of contralateral dehiscence in patients with unilaterally enlarged AC oVEMP amplitudes. This implies AC oVEMP sensitivity is not 100% and may on occasion miss smaller dehiscences. All cases with false negative AC oVEMP results had a CT classification score of 3, suggesting focal dehiscence in the short arm of the canal. Amplitudes and latencies to BC stimuli were also normal in these cases, implying a similar loss of sensitivity. Alternatively, the CT scans for some of these patients may have been classified as dehiscent in error. Even with 0.5 mm collimations, very thin bone can be invisible on CT imaging (23), leading to misdiagnosis of frank dehiscence in up to a third of near dehiscence cases (22). Over-diagnosis of frank dehiscence might also account for some cases in the dehiscence group that were without a BC oVEMP latency prolongation. More studies and case reports are needed to understand how often, and why, false negative AC oVEMPs might occur.

### Stimulus Considerations

While both BC stimuli used in this study produced significant latency effects, the effect size was largest for the lower frequency 125 Hz stimulus. Such low frequencies have been used infrequently for BC oVEMP testing and in guinea pigs with an intact bony labyrinth, they activate both irregular discharging otolith and canal afferents (27). We advocate its use, not as a test of otolith function, but as an adjunct test for diagnosing SCD. It is unknown whether differences in stimulus shaping, polarity and duration affect sensitivity and specificity of 125 Hz oVEMPs in SCD. The first studies involving 125 Hz stimulation in SCD used a 10 ms condensation polarity stimulus (2 ms rise/fall) (43, 44). Manzari et al. used a slightly shorter 7 ms 125 Hz stimulus, also of condensation polarity and although latencies were not analyzed, morphological changes (double-peaked configuration) like those reported here were evident in the recordings of a single patient (7). Other investigators have used an unshaped, single cycle (i.e., 8 ms) of either condensation (45) or rarefaction (16) polarity, each proving useful in diagnosing SCD based on different outcome measures. To some extent the choice of stimulus parameters will be influenced by the type of evoked potential system, many of which require at least one complete stimulus cycle.

### Study Limitations

A limitation of this study was that most patients did not undergo surgery to confirm their temporal bone status, meaning the possibility of misdiagnosed frank dehiscence could not be investigated. Other limitations of this study arise mainly from the retrospective design. Patients did not undergo cVEMP threshold testing since our clinic preferentially uses oVEMP for diagnosis of SCD, and those who were imaged at an alternate facility, were not represented. The number and range of alternate diagnoses in this study may differ from other centers and are likely to be influenced by clinic referral patterns and test protocols. For example, enlarged vestibular aqueducts may be less common in a neurology clinic compared with an ENT or audiology clinic, whereas vestibular migraine may be more common. Because we recruited patients based on an enlarged AC oVEMP, we are unable to compare the sensitivity of AC oVEMPs with BC oVEMP amplitudes and latencies. Prospective studies that recruit patients based solely on symptoms, and which are complemented by surgical confirmation of dehiscence, are needed to clarify the sensitivity and specificity of different VEMP outcome measures in SCD.

## Conclusions

Given the established high sensitivity of AC oVEMP amplitudes in SCD, we recommend these recordings continue to be prioritized as a first clinical test of dehiscence. However, as demonstrated in this study, AC oVEMPs can be enlarged for other reasons and in many cases, patients will fulfill symptom criteria for a dehiscence diagnosis. When this occurs, it is helpful to consider other test results. The demonstration of BC oVEMP latency delays in conjunction with an enlarged AC oVEMP amplitude are among the *ad hoc* indicators that can be considered. This may be particularly useful when CT imaging results and/or symptoms are ambiguous.

## Data Availability Statement

The raw data supporting the conclusions of this article will be made available by the authors, without undue reservation.

## Ethics Statement

The studies involving human participants were reviewed and approved by the Royal Prince Alfred Research Ethics and Governance Office. Written informed consent for participation was not required for this study in accordance with the national legislation and the institutional requirements.

## Author Contributions

RT and MW contributed to the conception and design of the study. JM interpreted the CT scans. RT, JM, BK, AY, BI, EA, NR, CR, JP, SR, and MW collected and/or collated data. RT performed the statistical analysis, prepared the figures and tables, and wrote the first manuscript draft. All authors contributed to manuscript revision, read, and approved the submitted version.

## Conflict of Interest

The authors declare that the research was conducted in the absence of any commercial or financial relationships that could be construed as a potential conflict of interest.

## Footnotes

^1^3 patients with bilaterally enlarged AC oVEMP due to frank dehiscence on one side and near dehiscence on the other, are included in the statistics for the dehiscent group.
